# Stories to Prevent Cancer: A Pilot Study Using Cancer Survivor Narratives to Increase Human Papillomavirus Vaccine Intentions

**DOI:** 10.1177/10732748241237328

**Published:** 2024-03-07

**Authors:** Chelsea M. Bufalini, Jennifer L. Kraschnewski, Timothy D. Riley, Kevin Wile, Katherine Spanos, Ashley Wong, Jessica Gall Myrick, Eric W. Schaefer, William A. Calo

**Affiliations:** 1 Department of Public Health Sciences, 12310 Penn State College of Medicine, Hershey, PA, USA; 2 Department of Medicine, 12310 Penn State College of Medicine, Hershey, PA, USA; 3 Department of Family and Community Medicine, 12310 Penn State College of Medicine, Hershey, PA, USA; 4 43963 George Washington University Hospital, Washington, DC, USA; 5 Bellisario College of Communications, 311370 Pennsylvania State University, University Park, PA, USA; 6 Penn State Cancer Institute, Hershey, PA, USA

**Keywords:** human papillomavirus vaccine, narratives, cancer prevention, adolescents, males

## Abstract

**Introduction:**

Human papillomavirus (HPV) vaccination rates are lower than other recommended adolescent vaccines. Cancer survivor narratives are used to promote cancer prevention and control, but little is known about their impact on adolescent HPV vaccination.

**Objective:**

This pilot study explored the feasibility and effects of a video education intervention using a cancer survivor narrative to improve parents’ attitudes toward and intentions to get the HPV vaccine.

**Methods:**

This study utilized a one-group design; participants completed a pre-intervention survey, watched the video before attending their sons’ wellness visits, and completed a post-intervention survey within one week of their appointment. Using the narrative persuasion framework, we developed a 4-minute video of a local HPV-related cancer survivor to promote the HPV vaccine as cancer prevention. We recruited 37 participants between June and October 2020. Participants were parents of males ages 9-17 who had not yet initiated HPV vaccination.

**Results:**

After the video, more parents agreed that HPV vaccination is safe (pre: 66% vs. post: 82%; *P* = .045) and that their child’s chances of getting HPV-related cancer in the future are high (pre: 24% vs. post: 46%; *P* = .014). Overall, 91% of parents felt the cancer survivor story helped them understand the risks of HPV cancers, and 52% said the story influenced their decision to start HPV vaccination for their child.

**Conclusions:**

Our findings suggest that cancer survivor narratives influence parents’ vaccine opinions and understanding of their child’s risk of HPV infection, leading to increased parental intent to get the HPV vaccine for their adolescent males.

## Introduction

Human papillomavirus (HPV)-attributable cancers affect more than 34,800 U.S. individuals annually.^1^ Because of this cancer burden, improving HPV vaccination rates is a national priority for cancer prevention.^2^ The Centers for Disease Control and Prevention’s (CDC) Advisory Committee on Immunization Practices (ACIP) recommends routine vaccination of the HPV vaccine at ages 11 or 12 years; however, vaccination can start as early as age 9. When initiated on time (ages 9-14), the HPV vaccine is a two-dose series and becomes a three-dose when given late (ages 15-26).^1^ Despite strong evidence supporting the safety and efficacy of the HPV vaccine in preventing six types of cancers and genital warts,^3-5^ only 60% of male and 64% of female adolescents aged 13 to 17 years old were up-to-date with HPV vaccination by 2021.^6^ This rate is far short of the Healthy People 2030 goal to have 80% of 13-15-year-olds up-to-date with the HPV vaccine.^7^ In the U.S., HPV vaccination rates remain lower than those of other adolescent vaccines, such as tetanus, diphtheria, and acellular pertussis vaccine (Tdap; 90%) and the meningococcal conjugate vaccine (89%).^6^ Low levels of HPV vaccination leave many of today’s U.S. children at unnecessary risk of morbidity and mortality caused by HPV-related cancers.^8^ Strong provider recommendation is the most powerful strategy for improving HPV vaccine rates.^9^ However, some parents express that they feel rushed to make vaccine decisions during clinic visits, their questions are not adequately answered,^10^ or they want additional information. To address this problem, it is necessary to identify influential messengers and effective messages that can supplement providers’ HPV vaccine communication.

Narrative communication is a promising approach to engage with individuals who are less involved, more resistant, in the early stages of behavior adoption, or have little knowledge about a health topic.^11,12^ This is especially important for HPV vaccination, as many parents lack knowledge of the vaccine or are resistant to vaccination due to concerns about side effects and an unproven association with increased sexual activity.^13,14^ Cancer survivors can play an important role in HPV vaccine education as messengers of accurate information and advocates for cancer prevention.^15^ Cancer survivor narratives are widely used in cancer prevention and education programs.^16,17^ Studies have shown that interventions in which survivors narrate their cancer experience and encourage people to undergo cancer screening are more effective than didactic approaches to increase mammography screening.^18^ These successes raise the possibility that using cancer survivor narratives will increase parents’ intentions to get the HPV vaccine for their unvaccinated children.

Health communication scholars have identified key elements of effective narratives, including narrative quality and message strength. For instance, narratives possessing elements of narrative quality (eg, plot and character development, emotional power and range, coherence, realism, and effective use of imagery^17^) may assist in motivating parents to get the HPV vaccine for their children. In the case of message strength, narratives that include explicit recommendations for HPV vaccination and repeat messages in favor of vaccination may be more persuasive than those that do not. Similarly, narratives that model a desired behavior (eg, seeking vaccination) and the resulting benefits (eg, prevention of HPV-related cancers) or demonstrate how barriers to a behavior can be overcome (eg, easing parents’ concerns about the HPV vaccine) should increase the motivation of audiences to engage in the same behavior. Unfortunately, narratives employing these elements of narrative quality and message strength are vastly underused for HPV vaccine education.^19^ Also, there is little evidence on how narratives of cancer survivors diagnosed with HPV-related cancer are received in the context of adolescent HPV vaccination.

Our project aimed to assess the effects of a video education intervention of cancer survivor stories to improve parents’ attitudes toward and intentions to initiate HPV vaccination among unvaccinated males aged 9-17. We focused on boys because they have lower HPV vaccination rates than girls in Pennsylvania and nationwide.^6^ We hypothesized that parents’ attitudes and intentions to initiate the HPV vaccine for their children would increase after watching our brief video intervention compared to their pre-intervention responses. We also explored the feasibility of using primary care clinics' existing workflow and digital communication systems to recruit for this study.

## Methods

### Participants and Procedures

This pilot study used a one-group design; participants received the same video intervention and were asked to answer two brief online surveys before and after watching the video. Participants were recruited from June through October 2020 from three primary care clinics and one specialty clinic affiliated with Penn State Health in Hershey, Pennsylvania. All partnering clinics offer HPV vaccination to adolescent patients. We pre-screened potential participants using the healthcare system’s electronic medical records (EMR). We searched for males between 9 and 17 years of age, who had not initiated the HPV vaccine series, and had a scheduled appointment at any of the partnering clinics within the next two weeks. We invited the parents of those children who met the initial eligibility criteria through three recruitment modes: a message through the patient portal, a personalized phone call, and/or a mailed postcard. The type of recruitment mode used depended on the information available in the EMR. When possible, we used two recruitment modes with each parent. Our recruitment materials directed parents to REDCap,^20^ a secure data capture application for research studies, where they were presented with the summary explanation of research and gave their implied consent by completing a brief screener. To be eligible to participate, parents confirmed they were 18 years of age or older, had a male child between 9 and 17 years of age that had not initiated the HPV vaccine series, received care from one of the participating clinics, could fluently speak and read English, and had a valid email address.

A total of 507 unique individuals were identified during the pre-screening process. Sixty-five parents completed the online screener; among them, 42 were eligible to participate. Parents often screened ineligible due to not receiving primary care at a participating clinic or inaccuracies in the EMR regarding their vaccination status. Thirty-seven parents eventually enrolled in the study (our analytic sample). Study participants completed a baseline survey and watched the intervention video before their child’s clinic appointment. Parents then completed the post-intervention survey within three days after the clinic visit. All parts of the study occurred electronically using participants’ digital devices. Survey data was captured using a secure electronic survey platform (REDCap).^21^ Participants received $30 in gift cards for completing both surveys. The Pennsylvania State University Institutional Review Board reviewed and approved the study (Hershey, PA, USA; May 14, 2020; STUDY00014531).

### Intervention

The intervention was a 4-minute video that featured a real male cancer survivor narrating his experience with HPV-related oropharyngeal cancer and recommending HPV vaccination. The story was created by applying the Narrative Persuasion Framework’s focus on including attributes of narrative quality and message strength.^17^ For example, the video included the following attributes: a plot, character development (ie, the survivor’s changes throughout his experience), emotional power (ranging from the survivor being overwhelmed to feeling despair during the cancer treatment), coherence of the story, realism, and effective use of imagery (eg, photographs of the survivor’s physical change from before to after cancer treatment). The video began with the survivor stating his name and the Pennsylvania town of residence and narrating his experience receiving a cancer diagnosis. The story continued with details about his treatment, including its impact on his life and family. The end of the story focused on message strength, with the survivor acknowledging the HPV vaccine, which was not available when he was a kid, and strongly recommended that parents vaccinate their male children to prevent the future development of HPV-related cancer. The video ended with a closing graphic that read, “HPV vaccine is cancer prevention.”

A production professional recorded the cancer survivor story. A total of 25 minutes of footage was recorded. The footage was carefully reviewed as the study team compiled time clips within the survivor’s footage to produce a story following the narrative persuasion framework. Then, the video production company created a 4-minute film, and we went through three revision cycles to refine the transitions between clips and opening and closing graphics. The intervention video and similar videos are available online at https://ctsi.psu.edu/isc/resources/.

### Measures

#### Vaccine attitudes

Both pre-and post-intervention surveys asked parents about their attitudes toward general vaccines,^22^ including their agreement with statements, “Vaccines do a good job in preventing the diseases they are intended to prevent” and “Vaccines are safe.” The surveys also included several items aligned with Health Belief Model (HBM) constructs, such as susceptibility, perceived benefits, and perceived barriers.^23^ We asked about susceptibility with the statement, “My child’s chances of getting HPV cancer in the future are great.” The perceived benefit was assessed with one item, “Having the HPV vaccine will help my child prevent HPV cancers.” Barriers to HPV vaccination included two items, “The HPV vaccine is being pushed to make money for drug companies” and “The HPV vaccine might cause my child health problems in the future.” All items used a 5-point response scale ranging from “strongly disagree” to “strongly agree.” Finally, we assessed anticipated regret, a cognitive emotion many experience when realizing or imagining that a situation would have been better if only a different decision had been made.^24^ This was assessed by providing a brief scenario to parents where their child got an HPV infection and subsequent cancer that the vaccine may have prevented, then asking, “How much would you regret that your child did not get the vaccine?” The 5-point response scale ranged from “not at all” to “quite a lot.”

#### Story Transportation and Believability

Transportation theory posits that a story invites the audience into the action it portrays through empathy for the story characters and imagination of the plot.^25^ The post-intervention survey assessed transportation with one item, “I could picture my child in the events described in the cancer survivor’s story.” The believability of narratives also plays a major role in an audience’s comprehension of story-based information and motivation to action.^26^ Believability consists of four constructs: story coverage (the degree to which a story accounts for all information presented), consistency (presence or absence of internal contradictions within a story), plausibility (judgment of the similarity of the story with other true or believable stories) and completeness (whether a story conforms to expectations about the way stories should be structured and organized). The post-intervention survey assessed believability with multiple items, including whether parents found the story to be true, easy to follow from beginning to end, consistent, relevant to parents, and helpful in understanding the risks of HPV cancers. These items used a 5-point response scale ranging from “strongly disagree” to “strongly agree.”

#### HPV Vaccine Intention

The post-survey also asked if the information from the story influenced their decision to get the HPV vaccine for their son (yes/no). We also asked parents whether they talked with their child’s healthcare provider about the information presented on the video intervention (yes/no).

#### Demographic Characteristics

The baseline survey assessed parents’ age, sex, race/ethnicity, and education level. We also reported the age of the child.

### Statistical Analysis

We reported descriptive statistics for all survey data. We used McNemar’s test to determine statistically significant changes between time points to assess the effect of the video intervention between the pre- and post-intervention surveys. For ease of interpretation, all questions originally asked with a 5-point agreement scale were dichotomized to “Disagree/Neither” and “Agree,” and responses to the anticipated regret item were dichotomized to “Not at all/A little/A moderate amount” and “Quite a lot.” All analyses were performed using SAS version 9.4 and R version 4.0.4.

## Results

The sample included 37 parents. All participants completed the pre-intervention survey, and 33 completed the post-intervention survey. Most participating parents were female (95%), ≥40 years of age (62%), non-Hispanic White (84%), had a bachelor’s degree or higher (51%), and reported on a child between 9 and 12 years of age (76%) (Table 1). After watching the video intervention, more parents agreed that vaccines are safe (pre: 66% vs. post: 82%; *P* = .045) and their child’s chances of getting HPV-related cancer in the future are high (pre: 24% vs. post: 46%; *P* = .014) (Table 2). Though not statistically significant (*P* > .05), our data also showed an increase in parents’ beliefs that the HPV vaccine is effective in preventing some cancers (pre: 65% vs. post: 79%; *P* = .16) and that parents would regret “quite a lot” if their child did not receive the vaccine but get cancer later in life (pre: 57% vs. post: 76%; *P* = .13).

**Table 1.** Participant Characteristics (n = 37).

	n (%)
Gender	
Male	2 (5)
Female	35 (95)
Age, years	
18-39	14 (38)
≥40	23 (62)
Race/Ethnicity	
Non-Hispanic White	31 (84)
Other	6 (16)
Education	
High school degree or equivalent	9 (24)
Associates degree	9 (24)
Bachelor’s or graduate degree	19 (51)
Age of child, years	
9-10	15 (41)
11-12	13 (35)
13-17	9 (24)

**Table 2.** Changes in Vaccine Attitudes Among Parents.

	Pre-intervention n (%)	Post-intervention n (%)	*P-*value
Vaccines do a good job in preventing the diseases they are intended to prevent			
Disagree/Neither	1 (3)	0 (0)	.32
Agree	35 (97)	33 (100)	
Vaccines are safe			
Disagree/Neither	12 (34)	6 (18)	.045*
Agree	23 (66)	27 (82)	
My child’s chances of getting an HPV cancer in the future are great			
Disagree/Neither	28 (76)	18 (54)	.014*
Agree	9 (24)	15 (46)	
The HPV vaccine is effective in preventing some cancers			
Disagree/Neither	13 (35)	7 (21)	.157
Agree	24 (65)	26 (79)	
Having the HPV vaccine will help my child prevent HPV cancers			
Disagree/Neither	15 (40)	8 (24)	.180
Agree	22 (60)	25 (76)	
The HPV vaccine is being pushed to make money for drug companies			
Disagree/Neither	24 (65%)	24 (73%)	.257
Agree	13 (35%)	9 (27%)	
The HPV vaccine might cause my child health problems in the future			
Disagree/Neither	22 (60%)	21 (64%)	.414
Agree	15 (40%)	12 (36%)	
How much would you regret that your child did not get the HPV vaccine?			
Not at all/A little/A moderate amount	16 (43)	8 (24)	.13
Quite a lot	21 (57)	25 (76)	

Regarding believability, most parents expressed that they believed the story to be true (94%), easy to follow from beginning to end (97%), helpful to understand the risks of HPV cancers (91%), relevant to parents (85%), and consistent (91%) (Table 3). Sixty-one percent of participants said they could picture their child in the story’s events, an indicator of story transportation. Nineteen percent of parents reported talking with their child’s healthcare provider about the information they saw on the video intervention. Half of the participants (52%) said watching the story influenced their decision to initiate HPV vaccination for their child.

**Table 3.** Narrative Transportation and Believability Ratings on the Cancer Survivor Story.

	n (%)
I could picture my child in the events described in the story	
Disagree/Neither	13 (39)
Agree	20 (61)
I believe the story to be true	
Disagree/Neither	2 (6)
Agree	31 (94)
The story was easy to follow from beginning to end	
Disagree/Neither	1 (3)
Agree	32 (97)
The story was helpful to understand the risks of HPV cancers	
Disagree/Neither	3 (9)
Agree	30 (91)
The information presented in the story was consistent	
Disagree/Neither	3 (9)
Agree	29 (91)
The events in the story are relevant to parents	
Disagree/Neither	5 (15)
Agree	28 (85)

## Discussion

In our pilot study, a brief video featuring a cancer survivor narrative influenced parents’ attitudes about vaccine safety and understanding of their child’s risk of HPV infection, with half of the participants reporting that our educational intervention influenced their decision to start HPV vaccination for their children. Our findings are consistent with a growing literature showing that narratives depicting personal experiences with HPV diseases increase a recipient’s perceived risk of infection, intentions to vaccinate, and decision-making toward HPV vaccination.^19^ Equally important, compared to prior studies testing printed narratives,^27,28^ our naturalistic videos provided stronger message persuasiveness and acceptability, as shown by the high ratings we obtained for believability and transportation. Of digital technologies, video consumption is the most rapidly growing area of mass communication, allowing us to better connect cancer prevention messaging with intended audiences.^29^

In the present study, we used existing consumer-centered technology, like patient portals and mobile devices, to deliver our educational video intervention remotely before in-person clinic visits. Our approach may be impactful for clinic-level HPV vaccination efforts because over one-fourth of providers inconsistently recommend the HPV vaccine or do not recommend it at all.^9^ Also, time constraints during clinic visits limit parents’ opportunities to discuss and make HPV vaccination decisions. These situations undermine parents’ understanding and confidence in HPV vaccination. Offering pre-visit education via convenient digital media to parents could complement provider communication and increase HPV vaccine uptake. Our prior survey research of a national sample of 1,109 parents of adolescents, ages 11-17, indicates that 56% of parents want HPV vaccine information before their child’s wellness visit, guiding our study’s approach (manuscript in preparation). Therefore, digital interventions using cancer survivor narratives delivered before clinic visits can address parents’ communication needs and preferences for HPV vaccine information.

While most parents from our study reported high scores for believability and transportation, we could not evaluate these variables as potential mediators due to the design limitations of this being a pilot study. A systematic review of narrative interventions raised concerns that using narratives without understanding their mechanisms could influence the quality of decisions made by the consumer.^30^ For instance, there is evidence for transportation as a mechanism of narrative influence as it strongly predicts changes in knowledge, attitudes, and behaviors for skin cancer prevention^31^ and cervical cancer screening.^32^ Identification with the main character, another potential mechanism of influence, was not assessed in this study. Our study used the story of a male survivor of an HPV-related oropharyngeal cancer to persuade parents to vaccinate their sons. Any identification parents experienced in our study was likely facilitated by the matched gender and that the cancer survivor is from a neighboring community, allowing them to envision their son in the survivor’s story. Future studies should examine mediating psychological processes to understand how narratives influence HPV vaccination decisions, especially discrete negative emotions (eg, anticipated regret) and positive emotions (eg, hope). Prior studies have shown that the audience’s emotions experienced in response to narrative cancer prevention messages are some of the best predictors of behavioral intention.^33^ Hope, in particular, can be a very motivating emotion in prompting people to pursue cancer prevention behaviors.^34,35^

This study’s strengths include the professional video recording of real cancer survivor stories, developing a theory-guided intervention, and integrating our intervention into the clinics’ technology ecosystem and workflow before the clinical visit. This study also had limitations. First, despite conducting the study during typical peak vaccination time, because of COVID-19, many families missed or canceled their scheduled visits, which is reflected in our limited sample size. Also, there were some recruitment challenges related to inaccurate contact information in the EMR, including incorrect mailing addresses and disconnected phone numbers. This study explored the feasibility of using clinics’ existing digital communication systems for recruitment; however, at the time of this study (Jun-Oct 2020), only 31% of pre-screened eligible patients had a patient portal account, so we needed multiple recruitment modes. Additionally, we only recruited from clinics within a single health system, but this approach was necessary to maintain the fidelity of the intervention delivery and aid the interpretability of the results. This pilot study did not include power calculations, and the resulting sampling frame and small sample size limit the generalizability of our findings. Future large-scale research is needed to evaluate the impact of narrative communication delivered through clinics’ digital systems on clinical outcomes related to HPV vaccination. For instance, a randomized controlled trial (RCT) could randomize parents of unvaccinated children to our brief video intervention showcasing local cancer survivors narrating their experiences with an HPV-associated cancer and recommending the HPV vaccine or control and then, assess HPV vaccine initiation (≥1 dose) among participants at the time of the wellness visit. An RCT could also examine theory-based mediating psychological processes (cognitive and emotional) to better understand how narratives exert influence in the context of HPV vaccine decision-making.

## Conclusion

Our findings suggest that cancer survivor narratives influence parents’ vaccine opinions and understanding of their children’s risks of HPV infection, leading to increased intent to vaccinate their male children against HPV. Presenting videos of cancer survivors narrating their stories and recommending HPV vaccination before children’s wellness visits can help parents better contextualize cancer prevention messaging and complement providers’ HPV vaccine recommendations.

## Data Availability

The dataset used during the current study is available from the corresponding author upon reasonable request.

## References

[1] Senkomago V Henley SJ Thomas CC Mix JM Markowitz LE Saraiya M . Human papillomavirus-attributable cancers - United States, 2012-2016. MMWR Morb Mortal Wkly Rep. 2019;68(33):724-728. doi:10.15585/mmwr.mm6833a3 31437140 PMC6705893

[2] The President’s Cancer Panel . HPV Vaccination for Cancer Prevention: Progress, Opportunities, and a Renewed Call to Action. Bethesda, MD: The President’s Cancer Panel; 2018.

[3] Phillips A Patel C Pillsbury A Brotherton J Macartney K . Safety of human papillomavirus vaccines: an updated review. Drug Saf. 2018;41(4):329-346. doi:10.1007/s40264-017-0625-z 29280070

[4] Huh WK Joura EA Giuliano AR , et al. Final efficacy, immunogenicity, and safety analyses of a nine-valent human papillomavirus vaccine in women aged 16-26 years: a randomised, double-blind trial. Lancet. 2017;390(10108):2143-2159. doi:10.1016/S0140-6736(17)31821-4 28886907

[5] Herrero R Quint W Hildesheim A , et al. Reduced prevalence of oral human papillomavirus (HPV) 4 years after bivalent HPV vaccination in a randomized clinical trial in Costa Rica. PLoS One. 2013;8(7):e68329. doi:10.1371/journal.pone.0068329 23873171 PMC3714284

[6] Pingali C Yankey D Elam-Evans L , et al. National vaccination coverage among adolescents aged 13-17 Years - national immunization survey-teen, United States, 2021. MMWR Morb Mortal Wkly Rep. 2022;71(35):1101-1108. doi:10.15585/mmwr.mm7135a1 36048724 PMC9472778

[7] ODPHP . Increase the proportion of adolescents who get recommended doses of the HPV vaccine — IID08. Healthy People 2030. https://health.gov/healthypeople/objectives-and-data/browse-objectives/vaccination/increase-proportion-adolescents-who-get-recommended-doses-hpv-vaccine-iid-08. Accessed March 25, 2021.

[8] The President’s Cancer Panel . Accelerating HPV Vaccine Uptake: Urgency for Action to Prevent Cancer. Bethesda, MD: The President’s Cancer Panel; 2014.

[9] Gilkey MB Calo WA Moss JL Shah PD Marciniak MW Brewer NT . Provider communication and HPV vaccination: the impact of recommendation quality. Vaccine. 2016;34(9):1187-1192. doi:10.1016/j.vaccine.2016.01.023 26812078 PMC4944755

[10] Shah PD Calo WA Gilkey MB , et al. Questions and concerns about HPV vaccine: a communication experiment. Pediatrics. 2018;143(2):e20181872. doi:10.1542/peds.2018-1872 PMC6361359 30670584

[11] McQueen A Kreuter MW Kalesan B Alcaraz KI . Understanding narrative effects: the impact of breast cancer survivor stories on message processing, attitudes, and beliefs among African American women. Health Psychol. 2011;30(6):674-682. doi:10.1037/a0025395 21895370 PMC3217077

[12] Moyer-Gusé E Nabi RL . Explaining the effects of narrative in an entertainment television program: overcoming resistance to persuasion. Health Commun Res. 2010;36(1):26-52. doi:10.1111/j.1468-2958.2009.01367.x

[13] Dilley SE Peral S Straughn JM Scarinci IC . The challenge of HPV vaccination uptake and opportunities for solutions: lessons learned from Alabama. Prev Med. 2018;113:124-131. doi:10.1016/j.ypmed.2018.05.021 29800594 PMC8863498

[14] VanWormer JJ Bendixsen CG Vickers ER , et al. Association between parent attitudes and receipt of human papillomavirus vaccine in adolescents. BMC Publ Health. 2017;17(1):1-7. doi:10.1186/s12889-017-4787-5 PMC5625818 28969653

[15] Mcqueen A Kreuter MW Kalesan B Alcaraz KI . Understanding narrative effects: the impact of breast cancer survivor stories on message processing, attitudes, and beliefs among African American women. Health Psychol. 2011;30(6):674-682. doi:10.1037/a0025395.Understanding 21895370 PMC3217077

[16] Perez M Sefko J Ksiazek D , et al. A novel intervention using interactive technology and personal narratives to reduce cancer disparaities: African American breast cancer survivor stories. J Cancer Surviv. 2014;8(1):21-30. doi:10.1007/s11764-013-0308-4 24030573 PMC3945406

[17] Kreuter MW Green MC Cappella JN , et al. Narrative communication in cancer prevention and control: a framework to guide research and application. Ann Behav Med. 2007;33(3):221-235. doi:10.1007/BF02879904 17600449

[18] Kreuter MW Holmes K Alcaraz K Richert M Mcqueen A Clark EM . Comparing narrative and informational videos to increase mammography in low-income African American women. Patient Educ Couns. 2010;81(Suppl: S6-14):1-19. doi:10.1016/j.pec.2010.09.008.Comparing 21071167 PMC3146295

[19] Okuhara T Kagawa Y Okada H Tsunezumi A Kiuchi T . Intervention studies to encourage HPV vaccination using narrative: a scoping review. Patient Educ Counsel. 2023;111:107689. doi:10.1016/j.pec.2023.107689 36868003

[20] Harris PA Taylor R Thielke R Payne J Gonzalez N Conde JG . Research electronic data capture (REDCap) – A metadata-driven methodology and workflow process for providing translational research informatics support. J Biomed Inf. 2009;42(2):377-381. 10.1016/j.jbi.2008.08.010 PMC2700030 18929686

[21] Harris PA Taylor R Thielke R Payne J Gonzalez N Conde JG . Research electronic data capture (REDCap) – A metadata-driven methodology and workflow process for providing translational research informatics support. J Biomed Inf. 2009;42(2):377-381. 10.1016/j.jbi.2008.08.010 PMC2700030 18929686

[22] Gilkey MB Magnus BE Reiter PL McRee AL Dempsey AF Brewer NT . The Vaccination Confidence Scale: a brief measure of parents’ vaccination beliefs. Vaccine. 2014;32(47):6259-6265. doi:10.1016/j.vaccine.2014.09.007 25258098 PMC4418546

[23] Champion VL . Revised susceptibility, benefits, and barriers scale for mammography screening. Res Nurs Health. 1999;22(4):341-348. doi:10.1002/(SICI)1098-240X(199908)22:4<341::AID-NUR8>3.0.CO;2-P 10435551

[24] Leder S Florack A Keller J . Self-regulation and protective health behaviour: how regulatory focus and anticipated regret are related to vaccination decisions. Psychol Health. 2015;30(2):165-188. doi:10.1080/08870446.2014.954574 25137215

[25] Green MC Brock TC . The role of transportation in the persuasiveness of public narratives. J Pers Soc Psychol. 2000;79(5):701-721. doi:10.1037/0022-3514.79.5.701 11079236

[26] Yale RN . Measuring narrative believability: development and validation of the Narrative Believability Scale (NBS-12). J Commun. 2013;63(3):578-599. doi:10.1111/jcom.12035

[27] Krakow M Yale R Perez DT Christy K Jensen J . Death narratives and cervical cancer: impact of character death on narrative processing and HPV vaccination. Health Psychol. 2017;36(12):1173-1180. doi:10.1037/hea0000498 28749148

[28] Hopfer S . Effects of a narrative HPV vaccination intervention aimed at reaching college women: a randomized controlled trial. Prev Sci. 2012;13(2):173-182. doi:10.1007/s11121-011-0254-1 21993613

[29] Finkler W Leon B . The power of storytelling and video: a visual rhetoric for science communication. J Sci Commun. 2019;18(5):A02. doi:10.22323/2.18050202

[30] Winterbottom A Bekker HL Conner M Mooney A . Does narrative information bias individual’s decision making? A systematic review. Soc Sci Med. 2008;67(12):2079-2088. doi:10.1016/j.socscimed.2008.09.037 18951673

[31] Dillard AJ Ferrer RA Welch JD . Associations between narrative transportation, risk perception and behaviour intentions following narrative messages about skin cancer. Psychol Health. 2018;33(5):573-593. doi:10.1080/08870446.2017.1380811 28975805

[32] Murphy ST Frank LB Chatterjee JS Baezconde-Garbanati L . Narrative versus nonnarrative: the role of identification, transportation, and emotion in reducing health disparities. J Commun. 2013;63(1):116-137. doi:10.1111/jcom.12007 PMC3857102 24347679

[33] Myrick JG Oliver MB . Laughing and crying: mixed emotions, compassion, and the effectiveness of a YouTube PSA about skin cancer. Health Commun. 2015;30(8):820-829. doi:10.1080/10410236.2013.845729 24877892

[34] Nabi RL Myrick JG . Uplifting fear appeals: considering the role of hope in fear-based persuasive messages. Health Commun. 2019;34(4):463-474. doi:10.1080/10410236.2017.1422847 29313717

[35] Myrick JG . An experimental test of the roles of audience involvement and message frame in shaping public reactions to celebrity illness disclosures. Health Commun. 2019;34(9):1060-1068. doi:10.1080/10410236.2018.1461170 29652513

